# Bactericidal Mechanism of Chlorous Acid Water in the Inactivation of Non-Tuberculous Mycobacteria

**DOI:** 10.3390/ijms27104570

**Published:** 2026-05-19

**Authors:** Hitoshi Yamaoka, Haruyuki Nakayama-Imaohji, Hisashi Yamasaki, Ayano Tada, Isanori Horiuchi, Tamiko Nagao, Nafisa Tabassum, Emmanuel Munyeshyaka, Hisataka Goda, Tomomi Kuwahara

**Affiliations:** 1 Department of Microbiology, Faculty of Medicine, Kagawa University, 1750-1 Miki-Town, Kita-District, Takamatsu 761-0793, Kagawa, Japan; s18d733@kagawa-u.ac.jp (H.Y.); imaoji.haruyuki@kagawa-u.ac.jp (H.N.-I.); tada.ayano@kagawa-u.ac.jp (A.T.); nafisa.maisha94@gmail.com (N.T.); s22d730@kagawa-u.ac.jp (E.M.); 2 Sankei Co., Ltd., 12th Floor, Osaka-Tokyo Marine Nichido Building, 2-2-53 Shiromi, Chuo-Section, Osaka City 540-0001, Osaka, Japan; isanori.horiuchi@sankei-group.com (I.H.); sankei5731@sankei-group.com (H.G.); 3 Division of Biology, Hyogo Medical University, 1-1 Mukogawa-Town, Nishinomiya City 663-8501, Hyogo, Japan; hi-yamasaki@hyo-med.ac.jp; 4 Department of Science for Human Health Welfare Care Major, Shikoku University Junior College, 123-1 Ebisuno, Furukawa, Ojin-Town, Tokushima-Citya 771-1192, Tokushima, Japan; tnagao@shikoku-u.ac.jp

**Keywords:** chlorous acid water, sodium hypochlorite, bactericidal mechanism, *Mycobacterium avium* complex, DNA damage, membrane potential

## Abstract

The global prevalence of pulmonary infections caused by non-tuberculous Mycobacteria (NTM), particularly the *Mycobacterium avium* complex (MAC), is increasing. Since NTM are ubiquitous in moist environments and resistant to standard disinfectants, this study evaluated the efficacy of chlorous acid water (CAW) against them. CAW demonstrated superior sanitizing effects compared to sodium hypochlorite (NaClO), efficiently inactivating NTM at 100 mg/L free available chlorine even in the presence of organic matter, where 1000 mg/L NaClO failed. Instead, subcellular fractionation and protein analysis revealed that CAW penetrates the cell to induce extensive aggregation of internal functional proteins, leading to the rapid collapse of membrane potential and ATP production. Furthermore, CAW exhibited significantly lower cytotoxicity toward human lung-derived A549 cells than NaClO. These results indicate that CAW inactivates NTM effectively by targeting internal protein stability and the respiratory chain, offering a potent and safer disinfection strategy for clinical and domestic environments.

## 1. Introduction

In recent years, while the incidence of tuberculosis has been decreasing [1], the incidence of non-tuberculous mycobacteria (NTM) infections has been increasing [2]. Among the NTM, pulmonary infections by *Mycobacterium avium* complex (MAC) bacteria show the highest incidence and are becoming a serious concern for public health [3,4,5,6]. The main causative agents of pulmonary NTM infection are *M. avium* and *M. intracellulare* [7]. The prevalence of MAC isolated from pulmonary NTM infections shows a unique geographical distribution in Japan. *M. avium* is primarily isolated from pulmonary NTM infections in eastern Japan, whereas *M. intracellulare* is found in western Japan [7,8]. Pulmonary infections caused by *M. intracellulare* have a higher clinical morbidity than those caused by *M. avium* [9,10]. Pulmonary MAC infection is difficult to treat with antibiotics, and it requires long-term therapy (at least 1 year to achieve sputum culture negativity) with combinations of antibiotics, which include antituberculosis agents and macrolides [11]. Microbiological recurrence due to MAC reinfection is often observed even after successful clinical treatment [12,13].

NTM including MAC are widely distributed in the natural environment [14], and they are particularly abundant in moist or wet environments such as tap water and bathrooms [15]. Therefore, hygienic management of moist or wet environments is required to reduce exposure to NTM. Microbiological surveillance in the households of 49 pulmonary MAC disease outpatients and 43 healthy volunteers in Japan reported that MAC was isolated mainly from bathrooms and not from kitchen tap water, wash basins, and other sites [16]. In addition, the incidence of MAC was significantly higher in the bathrooms of patients than in those of healthy volunteers [14].

The rapid detection and eradication of pathogenic microorganisms from infection sources is essential to preventing infectious diseases. However, effective methods for eradicating NTM remain to be established. NTM shows relatively high resistance to chlorine-based disinfectants [17], and of the NTM species, MAC is the most tolerant to disinfectant [18]. Effective sanitizing methods for bathrooms, which are rich in biological substances of human origin and biofilm materials, are needed.

Recently, a chlorite (HClO_2_)-based disinfectant, chlorous acid water (CAW), has been approved as a Category II disinfectant in Japan. CAW has greater bactericidal effects under organic-matter-rich conditions when compared to sodium hypochlorite (NaClO), which is a representative chlorine-based disinfectant [19]. Therefore, CAW is a suitable disinfectant for environments contaminated by biological substances, which include food processing equipment and hospital environments that are contaminated by human excretions.

The aims of this study were to examine whether CAW has a sufficient bactericidal effect against *M. intracellulare* and to elucidate the bactericidal mechanism of this disinfectant against mycobacteria.

## 2. Results

### 2.1. Bactericidal Effects of CAW and NaClO on NTM

**Figure 1:**
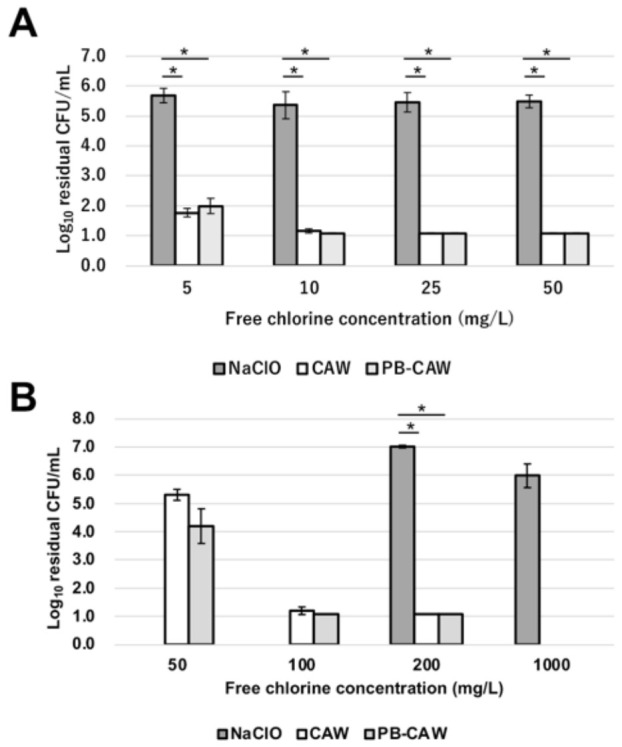
Reduction in viable *M. intracellulare* cells after 1 min of treatment with CAW, PB-CAW or NaClO. The bactericidal effect of each reagent was evaluated under organic-matter-free conditions (**A**) or in the presence of 0.5% BSA (**B**). The residual viable cell numbers/mL is shown on a logarithmic scale on the *y* axis. The data are expressed as means ± standard errors from three independent repeats. The differences in the log_10_ residual viable cell number/mL in *M. intracellulare* by the test disinfectants were statistically examined at each FAC by one-way ANOVA followed by Tukey’s test. Asterisks indicate statistically significant differences (* *p* < 0.05). FAC, free available chlorine; BSA, bovine serum albumin.

We examined the bactericidal effects of chlorous acid water (CAW), phosphate-buffered CAW (PB-CAW), and sodium hypochlorite (NaClO) on *Mycobacterium intracellulare* NBRC 112750. PB-CAW (pH 7–8) is produced by adjusting the pH of CAW (pH 5–6) with phosphate buffer (pH 7.4). As shown in Figure 1A, CAW and PB-CAW containing >5 mg/L free available chlorine (FAC) efficiently inactivated *M. intracellulare* within 1 min of treatment (>4.0 log_10_ reduction in CFU/mL) in the absence of organic-matter load. The log_10_ reduction in viable *M. intracellulare* by NaClO was <1.0 even with 200 mg/L. When bovine serum albumin (BSA) was added to the reaction (0.5% final concentration), the sanitizing effect of all the test reagents was reduced (Figure 1B). To achieve effective killing (defined as a >4.0 log_10_ reduction in CFU/mL) within 1 min, >100 mg/L was required for both CAW and PB-CAW. On the other hand, NaClO reduced the concentration of viable cells only by around 1.0 log_10_ CFU/mL even at 1000 mg/L in the presence of 0.5% BSA.

**Figure 2:**
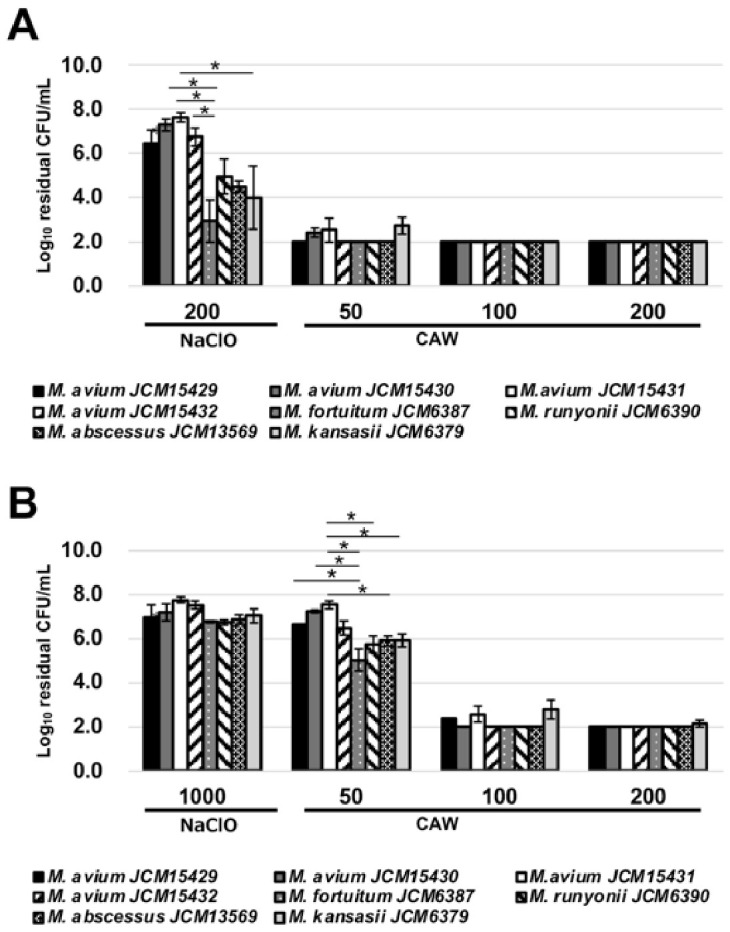
The reduction in viable mycobacterial strains other than *M. intracellulare* after a 1 min treatment with CAW or NaClO. The bactericidal effect of each reagent was evaluated in the absence of organic matter (**A**) or in the presence of 0.5% BSA (**B**). The numbers below the *x* axis indicate the FAC concentration in the reaction mixture. The residual viable cell numbers/mL is shown on a logarithmic scale on the *y* axis. The data are expressed as means ± standard errors from three independent repeats. The differences among the strains were statistically examined at each treatment condition by one-way ANOVA followed by Tukey’s test. Asterisks indicate statistically significant differences (* *p* < 0.05). FAC, free available chlorine; BSA, bovine serum albumin.

Bactericidal tests for other mycobacterial species (Table S1) were conducted under the same conditions employed for *M. intracellulare*. Although species-dependent differences in the sensitivity to test sanitizers were observed, CAW showed bactericidal effects on all the tested mycobacteria similar to the effect on *M. intracellulare*. A 1 min treatment with CAW containing >50 mg/L and >100 mg/L achieved efficient killing under organic-matter-free and 0.5% BSA-loading conditions, respectively (Figure 2A,B). *M. intracellulare* and *M. avium* were more resistant to NaClO than the other mycobacteria tested (Figure 1A and Figure 2A).

### 2.2. Effects of CAW and NaClO on the Morphology of Mycobacteria

**Figure 3:**
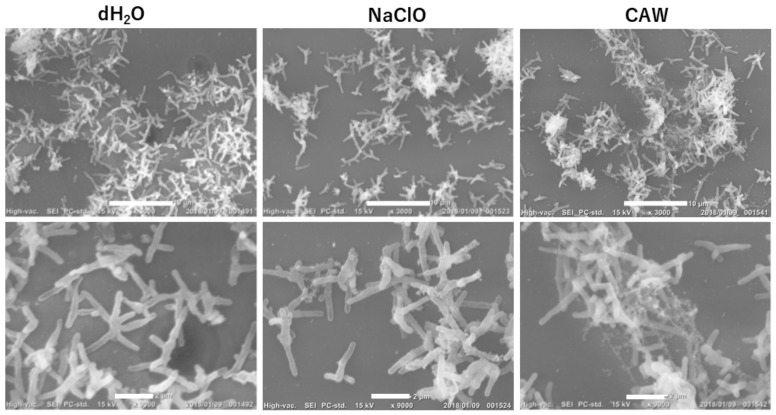
Scanning electron microscope images of *M. intracellulare* cells after 30 min of treatment with each agent. The upper and lower panels are images observed at 3000× and 9000× magnifications, respectively. The white scale bars in the upper and lower panels correspond to 10 μm and 2 μm, respectively.

To elucidate the bactericidal mechanisms of CAW and NaClO, *M. intracellulare* NBRC 112750 was treated with each agent at a FAC concentration of 200 mg/L for 30 min, and the subsequent bacterial surface structure was observed by scanning electron microscopy (SEM). After this treatment, the bacterial count of *M. intracellulare* was below the detection limit (1.08 log_10_ CFU/mL), indicating that it was completely inactivated by CAW, whereas no significant decrease in viability was observed in the sterile-water- and NaClO-treated groups. As shown in Figure 3, an extracellular matrix, which is a structure contributing to biofilm formation, was clearly observed around the *M. intracellulare* cells in the sterile water group. Although NaClO treatment eliminated the extracellular matrix, no change in cell morphology was observed in this group. Likewise, CAW treatment did not alter the *M. intracellulare* cell morphology, but the extracellular matrix shrank and agglutinated around the cells. These results suggest that the bactericidal effect of CAW may not be due to direct damage to the bacterial surface.

### 2.3. Effects of CAW, PB-CAW and NaClO on Mycobacterial Genomic DNA

**Figure 4:**
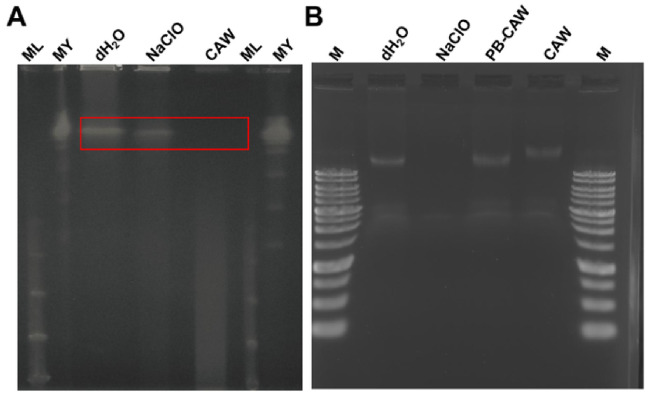
Degradation of genomic DNA from *M. intracellulare* after 30 min of treatment with the test reagents. (**A**) PFGE images of the genomic DNA from *M. intracellulare* after treatment with 200 mg/L each of NaClO or CAW for 30 min. The position of intact chromosomal DNA is shown by a red square. ML, lambda ladder marker; MY, yeast chromosome marker. (**B**) Agarose gel electrophoresis of the purified *M. intracellulare* genomic DNA with CAW, PB-CAW or NaClO for 1 min. M, 1 kb DNA ladder marker.

We evaluated the degradation of mycobacterial DNA after treatment with CAW or NaClO. *M. intracellulare* NBRC 112750 was exposed to each reagent at a FAC concentration of 200 mg/L for 30 min, followed by neutralization with 1 M sodium thiosulfate. As shown in Figure 4A, pulsed-field gel electrophoresis (PFGE) revealed that CAW treatment induced genomic DNA degradation in mycobacterial DNA, whereas NaClO treatment did not. To examine the direct action of CAW on DNA, we treated the purified genomic DNA from *M. intracellulare* NBRC 112750 with CAW, PB-CAW or NaClO under the same conditions. The results were contradictory to the PFGE analysis, in which CAW and PB-CAW did not break down the DNA, whereas NaClO did cause DNA degradation (Figure 4B). These results indicated that *M. intracellulare* DNA degradation did not result from the direct action of CAW, but from stress responses induced by the reagent.

Notably, the migration of the genomic DNA in the agarose gel was delayed by CAW treatment (200 mg/L, 30 min), while the PB-CAW (buffered around neutral pH with phosphate buffer) showed no effect (Figure 4B). S1 nuclease, which digests single-stranded DNA, largely degraded the genomic DNA after exposure to CAW (pH 5–6, unbuffered), which was not the case with the PB-CAW (Figure S1), indicating that CAW affects the hydrogen bonding pattern within double-strand DNA.

To determine whether DNA degradation is responsible for the bacterial killing by CAW, we extracted DNA from *M. intracellulare* NBRC 112750 and treated the DNA with each disinfectant at the FAC levels used in the bactericidal tests (5, 10, 25, 50, 100, and 200 mg/L) for 1 min. None of the disinfectants affected the genomic DNA (Figure S2). These results indicate that DNA degradation is not the direct bactericidal mechanism for CAW and PB-CAW.

### 2.4. Effect of CAW, PB-CAW and NaClO on Membrane Potential

We measured the membrane potential of *M. intracellulare* NBRC 112750 treated with each disinfectant by using the BacLight™ Bacterial Membrane Potential kit. The kit employs the fluorescent dye DiOC_2_(3), which permeates cells and normally exhibits green fluorescence. In cells with intact membrane potential, the dye polymerizes within the membrane and exhibits red fluorescence. By calculating the ratio of these fluorescence intensities (red/green ratio), it is possible to evaluate changes in membrane potential. A decoupling reagent, carbonylcyanide *m*-chlorophenyl-hydrazone (CCCP) and sterilized distilled water were used as positive and negative controls, respectively.

**Figure 5:**
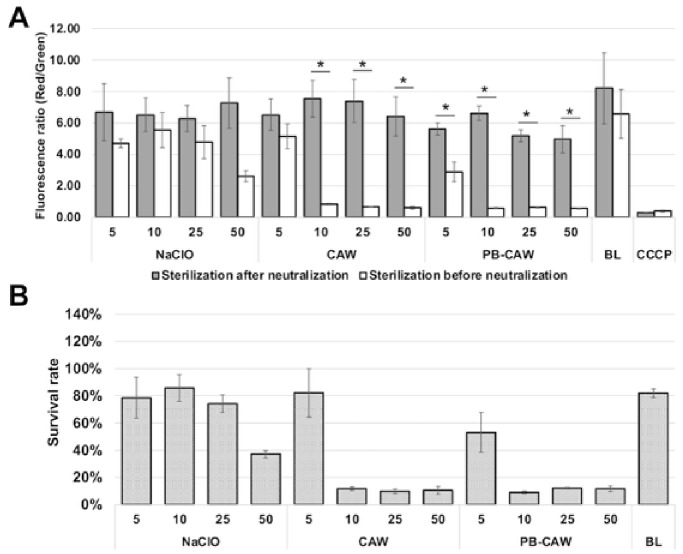
Effect of CAW, PB-CAW and NaClO on the membrane potential in *M. intracellulare*. (**A**) Ratio of red to green fluorescence after treatment with each reagent at varying FAC (mg/L) indicated below the *x*-axis (gray bars). Fluorescence from *M. intracellulare* cells that were inoculated into the pre-neutralized reagent with 1 M sodium thiosulfate was also measured to calculate the survival ratio (white bars). The difference in fluorescence ratios between treatments with pre-neutralized and non-neutralized disinfectants was statistically examined within the respective conditions using two-sample *t*-tests. Asterisks indicate statistically significant differences (* *p* < 0.05). (**B**) Survival rates after treatment with each reagent, which were calculated from the fluorescence ratios shown in panel A. The numbers below the *x*-axis indicate the FAC (mg/L) in the test solution. The data are expressed as means ± standard errors from three independent repeats. FAC, free available chlorine; CCCP, carbonyl cyanide *m*-chlorophenyl hydrazone; BL, blank (sterilized distilled water).

**Figure 6:**
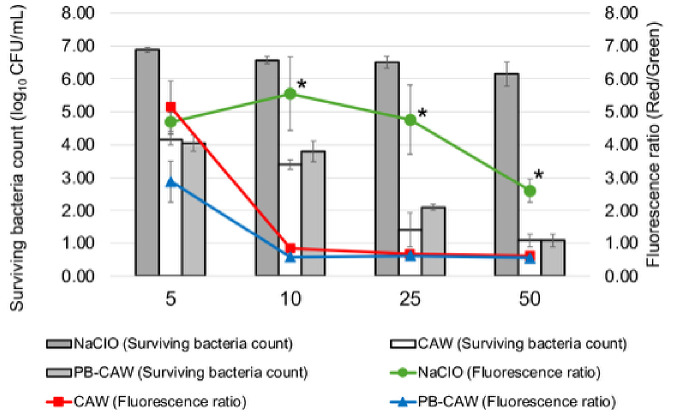
Correlation between the number of surviving *M. intracellulare* cells and the membrane potential after treatment with CAW, PB-CAW or NaClO. The FAC (mg/L) used is indicated below the *x*-axis. Bars and lines indicate the surviving cell number/mL on a logarithmic scale (log_10_) and the membrane potential inferred from the red/green fluorescence ratio. For each FAC (mg/L) used for treatment, a one-way ANOVA followed by Dunnett’s test was employed to examine the differences in the fluorescence ratios after treatment with NaClO or PB-CAW versus CAW treatment. Asterisks indicate statistically significant differences (* *p* < 0.05). FAC, free available chlorine.

Treatment with CAW and PB-CAW at >10 mg/L significantly reduced the membrane potential (Figure 5A) and the survival rate (Figure 5B) of *M. intracellulare* cells, while significant reductions in the membrane potential and survival rate were only observed at >50 mg/L after NaClO treatment. No significant reduction relative to the negative control was observed when *M. intracellulare* cells were inoculated with pre-neutralized reagents. These results indicate that CAW and PB-CAW disrupt the membrane potential in a similar manner, but at lower FAC, to NaClO (Figure 6).

### 2.5. Alterations in Intracellular ATP Levels After Treatment with CAW, PB-CAW and NaClO

**Figure 7:**
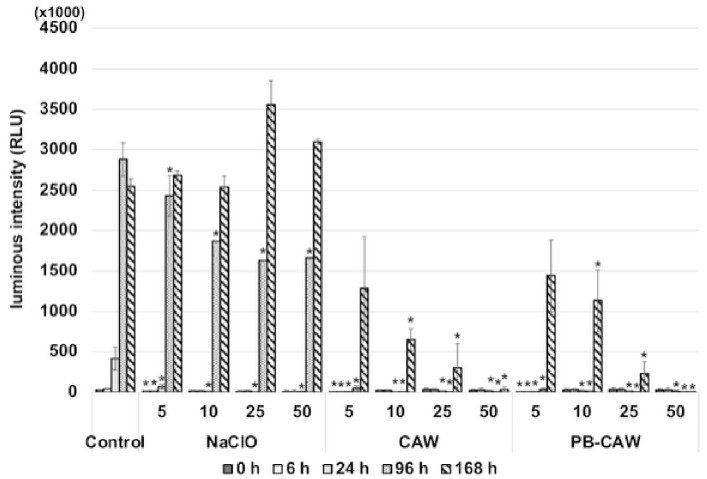
Recovery of ATP production in *M. intracellulare* after bactericidal treatment. The FAC concentration (mg/L) used is indicated below the *x*-axis. The data are expressed as means ± standard errors from three independent repeats. The control indicates the group that was not treated with any of the disinfectants. The time-dependent differences in the luminescence intensities were examined within each treatment condition (type of disinfectant and FAC) by a one-way ANOVA followed by Dunnett’s test (vs. 0 h). Asterisks indicate statistically significant differences (* *p* < 0.05).

To verify the membrane potential disruption by CAW or PB-CAW, we periodically measured ATP production using the BacTiter-Glo reagent and the GloMax Navigator system for a period of seven days after the treatment (Figure 7). After 24 h of treatment, ATP synthesis did not recover in any of the treated *M. intracellulare* cultures. However, after 7 days, ATP levels in the culture that was treated with NaClO at 5 to 50 mg/L recovered ATP production at levels equivalent to, or higher than, those of the control (25 and 50 mg/L). On the other hand, in the *M. intracellulare* cultures treated with CAW or PB-CAW, ATP production did not recover until 4 days after treatment. Recovery of ATP production was observed in the cells treated with CAW or PB-CAW at low FAC (5, 10, or 25 mg/L), but the level was lower than that of the control. The cells treated with CAW and the PB-CAW at 50 mg/L did not recover ATP production at all. These results indicate that CAW and the PB-CAW cause irreversible damage to the respiratory chain.

### 2.6. Reactive Oxygen Species (ROS) Generation After Treatment with Each Sanitizer

**Figure 8:**
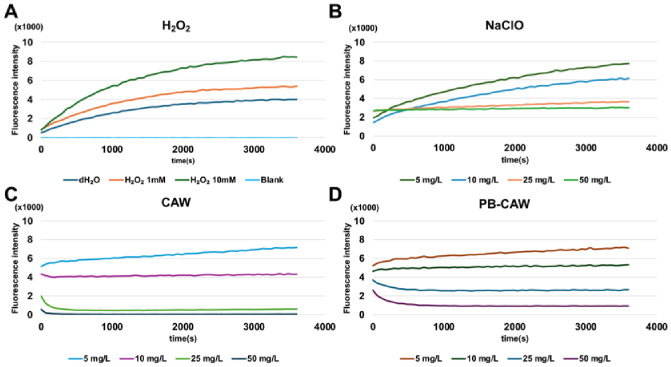
ROS production in *M. intracellulare* after treatment with bactericidal reagents. Fluorescence intensity was monitored every 1 min after *M. intracellulare* labeled with DCFA was exposed to each reagent: (**A**) H_2_O_2_ (1 and 10 mM), dH_2_O, or blank; (**B**) NaClO; (**C**) CAW; and (**D**) PB-CAW at concentrations of 5, 10, 25, and 50 mg/L.

Based on the results described above, we speculated that CAW and the PB-CAW caused severe damage to *M. intracellulare* membrane proteins, leading to dysfunction of the respiratory chain, loss of membrane potential and impairment of ATP synthesis. NADH dehydrogenase, which is a well-known constituent of the respiratory chain, is a major contributor to intracellular ROS production during redox reactions. Therefore, we measured intracellular ROS production in *M. intracellulare* after contact with the test reagents. We used hydrogen peroxide (H_2_O_2_) as a positive control to induce intracellular ROS production. H_2_O_2_ induced intracellular ROS production in a concentration-dependent manner (Figure 8A). In contrast, 25 mg/L and 50 mg/L CAW or PB-CAW decreased the intracellular ROS level (Figure 8B,C). On the other hand, 25 mg/L and 50 mg/L NaClO did not alter the intracellular ROS level (Figure 8D). At low FAC (5 mg/L and 10 mg/L), all reagents increased the intracellular ROS level, but a steeper curve was observed for NaClO than for CAW or the PB-CAW: the ratios of the last/initial fluorescence values were 6.4, 4.0, 1.4, and 1.4 for H_2_O_2_ (1 mM), NaClO (5 mg/L), CAW (5 mg/L) and PB-CAW (5 mg/L), respectively.

### 2.7. Impact of CAW on Bacterial Subcellular Protein Distribution and Aggregation Patterns

**Figure 9:**
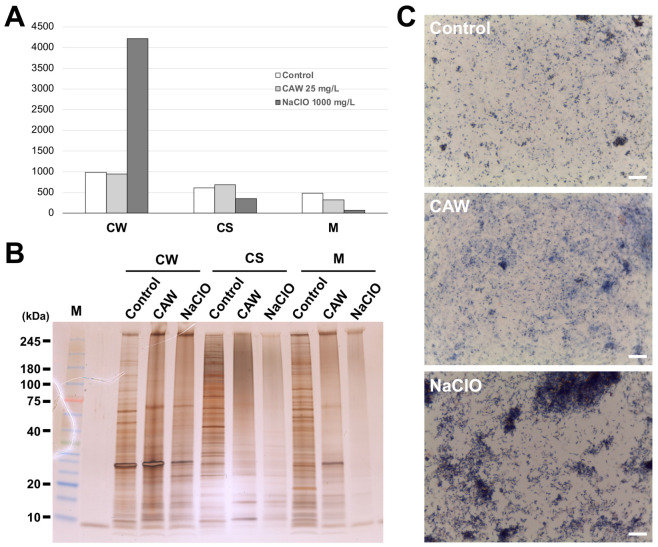
Impact of CAW and NaClO on subcellular protein distribution and aggregation in *M. intracellulare*. (**A**) Quantitative analysis of protein distribution in subcellular fractions. The protein concentration (μg/mL) in the cell wall (CW), cytosolic (CS), and membrane (M) fractions was determined by BCA assay after 1 min of treatment with sterile water (Control), 25 mg/L of CAW, or 1000 mg/L of NaClO. Data are presented as the mean of independent experiments. Note the massive increase in protein mass in the CW fraction of NaClO-treated cells, likely due to the entrapment of intracellular components within surface-level aggregates. (**B**) SDS-PAGE analysis of protein profiles. Proteins from each fraction (CW, CS, and M) were separated by SDS-PAGE and visualized by silver staining. The disappearance of distinct protein bands and the appearance of high-molecular-weight smears in CAW-treated CS and M fractions provide direct evidence of extensive protein denaturation and aggregation. (**C**) Morphological observation of the CW fraction. Gram staining (Favor G kit) was performed on the isolated CW fractions. While CAW-treated samples maintained a distribution pattern similar to the Control, NaClO treatment resulted in the formation of large, irregular clusters of cellular debris, indicating gross structural collapse and surface-level protein cross-linking. The white scale bars correspond to 10 μm. FAC, free available chlorine; CW, cell wall fraction; CS, cytosolic fraction; M, membrane fraction; SDS-PAGE, sodium dodecyl sulfate–polyacrylamide gel electrophoresis; BCA, bicinchoninic acid.

To elucidate the molecular targets of CAW, we performed subcellular fractionation and protein analysis of *M. intracellulare* treated with CAW or NaClO (Figure 9). Quantitative analysis of the protein distribution revealed that CAW treatment maintained protein levels in the cell wall (CW), cytosolic (CS), and membrane (M) fractions at levels comparable to those of the untreated control. In stark contrast, NaClO treatment led to a massive increase in protein mass within the CW fraction, with a concomitant decrease in the CS and M fractions. This suggests that the intense reaction of NaClO with the cell surface causes extensive cross-linking and the formation of large cellular aggregates, which likely reduced the extraction efficiency of internal proteins by trapping them within the insoluble CW debris. This result reinforces the conclusion that NaClO reacts predominantly and destructively with the cell surface, leading to gross structural collapse.

In contrast to NaClO, CAW-treated samples maintained total protein quantity across all fractions, yet SDS-PAGE demonstrated a dramatic shift in the qualitative protein profiles. In the CS and M fractions, distinct protein bands observed in the control group disappeared and were replaced by high-molecular-weight smears. This provides direct evidence that CAW penetrates the cell wall to reach internal compartments, where it induces the oxidative denaturation and physical aggregation of functional proteins without causing the immediate surface-level entrapment seen with NaClO. Furthermore, Gram staining of the CW fraction supported this, showing irregular clusters of cellular debris in NaClO-treated samples, while CAW-treated samples remained similar to the control. These findings indicate that while NaClO’s action is hindered by surface-level aggregation, CAW efficiently targets internal functional proteins, ultimately triggering the loss of membrane potential and ATP production.

### 2.8. Cytotoxicity of CAW on Human Lung-Derived A549 Cells

**Figure 10:**
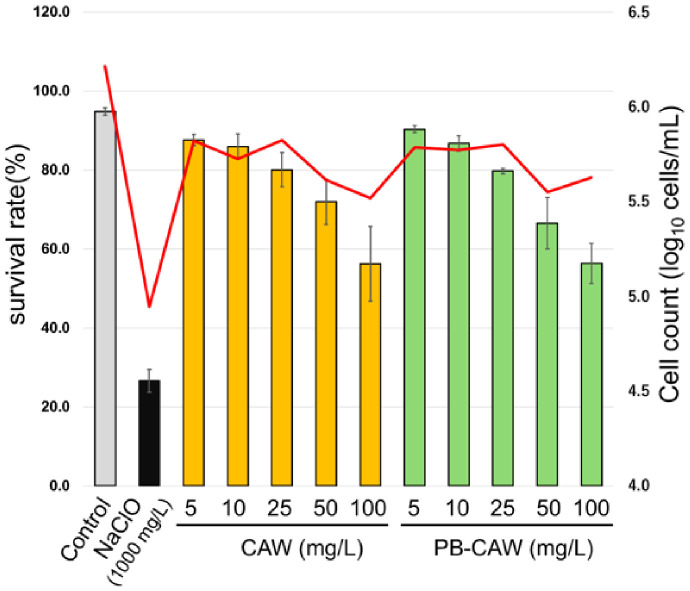
Cytotoxicity of CAW, PB-CAW, and NaClO on A549 cells. The viability of A549 human lung carcinoma cells (a model for alveolar epithelial cells) was assessed using the trypan blue exclusion method after 1 min of treatment with each disinfectant at the indicated FAC concentrations (mg/L). Bars represent the survival rate (%), and the red line indicates the total cell count (log_10_ cells/mL). Data are expressed as means ± standard errors from three independent repeats. FAC, free available chlorine.

To evaluate the safety profile of CAW, cytotoxicity assays were performed using the human lung-derived A549 cell line as a model for alveolar epithelial cells. As illustrated in Figure 10, A549 cells treated with CAW or PB-CAW at concentrations ranging from 5 to 100 mg/L exhibited high survival rates, with viability maintained between 56.4% and 90.3%. Even at 100 mg/L, the cell viability remained above 50%. Conversely, treatment with 1000 mg/L NaClO resulted in marked cytotoxicity, reducing the survival rate to 26.6%. These findings indicate that CAW possesses a lower toxicological risk to lung-derived cells compared to the high-concentration NaClO typically required for effective mycobacterial disinfection.

## 3. Discussion

Chlorite (HClO_2_)-based sanitizers, which include acidified sodium chlorite (ASC) and PB-CAW, are used for the sanitation of food and food processing facilities [20,21]. PB-CAW has recently been approved as a category 2 drug in Japan. The microbicidal activity of CAW against a wide spectrum of microorganisms, including *M. tuberculosis* [22], has been reported. These studies commonly reported that CAW showed microbicidal effects superior to those of NaClO under organic-matter-rich conditions. Furthermore, recent independent evaluation in clinical and biological settings has confirmed the high disinfection efficacy of CAW and deemed it a superior alternative to traditional sanitizers [23].

The incidence of pulmonary disease caused by MAC has been increasing in several countries where the incidence of tuberculosis is decreasing [1,2]. Moist and wet environments in households such as bathroom and kitchen sinks and water supply systems are reservoirs for NTM [15]. These environments are rich in organic matter derived from humans and from food ingredients, leading to biofilm and/or slime formation. Under these conditions, it is likely to be difficult to eliminate NTM using simple sanitation approaches. Therefore, we aimed to examine the microbicidal effect of CAW on NTM.

As in the previous study, we observed that CAW showed superior killing of NTM compared with NaClO at the same FAC level. Notably, MAC including *M. avium* and *M. intracellulare* showed higher resistance to NaClO than other NTM such as *M. fortuitum* and *M. abscessus* (Figure 2A). This indicates that chlorine levels in tap water are insufficient to inactivate NTM in water supply systems.

Even under organic matter loading conditions, CAW at >100 mg/L achieved a >4 log_10_ reduction in viable cells of all the tested NTM strains within 1 min of treatment, in contrast to NaClO, which did not achieve this level of reduction even at 1000 mg/L. The chloroperoxyl radical (ClOO˙) is a main active component of CAW [24]. This novel radical species can be detected by electron spin resonance without spin trap reagents, indicating that it is a long-lived radical. In addition, Goda et al. reported that CAW showed selective reactivity toward amino acids, reacting only with cysteine and histidine among the 15 amino acids tested [19]. These findings indicate that the tolerance of CAW to organic matter can be attributed to the long-lived chloroperoxyl radical and its selective reactivity. However, the microbicidal mechanisms of CAW have not been fully elucidated.

We first investigated whether the bactericidal mechanism of CAW was due to oxidative DNA damage. DNA purified from *M. intracellulare* was exposed to each of the FAC reagents at a concentration of 200 mg/L for 30 min. Contrary to the PFGE results, NaClO degraded the DNA band, whereas CAW and the CAW formulation did not reduce its intensity. However, CAW treatment resulted in an upward shift in the band. The shift in the DNA band observed after CAW treatment may suggest dissociation of double-stranded DNA (Figure 4B). This is likely due to disruption of hydrogen bonds between strands in double-stranded DNA, as predicted by experiments using S1 nuclease, which degrades single-stranded DNA (Figure S2). Furthermore, DNA was extracted after a 1 min contact time, during which CAW and the CAW formulation exerted efficient bactericidal activity. Although 200 mg/L NaClO reduced the intensity of the DNA band, DNA extracted from *M. intracellulare* remained unchanged after treatment with either reagent at 200 mg/L for 1 min (Figure S1).

Since there was no correlation between the DNA degradation and bactericidal effect of CAW, we concluded that DNA damage is not a primary bactericidal mechanism of CAW. Gupta et al. reported that exonucleases are induced in *Escherichia coli* cells by oxidative stress [25]. In the case of *M. intracellulare*, the DNA degradation shown in the PFGE analysis seemed to be a secondary phenomenon due to lethal oxidative stress induced by CAW. Transcriptome analysis will be needed to fully explain how physiological changes in NTM are induced by CAW. On the other hand, NaClO degraded purified DNA, but not DNA inside *M. intracellulare* cells, indicating that it is difficult for NaClO to penetrate the lipid-rich cell walls of mycobacteria. It was noteworthy that PB-CAW, which consists of CAW in phosphate buffer around neutral pH, did not degrade the DNA, but rather seemed to stabilize the DNA. This idea is based on the agarose gel image in which the smearing of DNA decreased after treatment with the PB-CAW (Figure S1). The reason for this effect is unknown, but the negative charges of the phosphate ion electrostatically interact with divalent cations such as Mg^2+^, which is a cofactor enhancing DNase activity. The pH adjustment of CAW with phosphate buffer may decrease the genotoxic effects of CAW or other oxidative sanitizers.

Hatanaka et al. reported that CAW agglutinated membrane proteins without affecting the genomic DNA, indicating that protein degeneration is a major bactericidal mechanism [26]. Accordingly, we measured the membrane potential, and the results showed that the membrane potential declines in a manner that correlates well with the bactericidal effects of CAW and PB-CAW on NTM. The reduction in membrane potential by CAW was attributed to the deterioration of respiratory chain function. CAW and PB-CAW were considered to cause irreversible degeneration of membrane proteins since ATP synthesis did not recover after treatment with CAW or PB-CAW at 50 mg/L, even seven days after treatment. On the other hand, *M. intracellulare* treated with NaClO recovered ATP synthesis, including higher ATP production than the control when the cells were treated with NaClO at 25 mg/L and 50 mg/L, indicating a compensatory response to increased energy demand for recovery from cellular damage.

Our subcellular fractionation and protein analysis (Figure 9) provided crucial insights into the distinct modes of action between CAW and NaClO. While NaClO treatment resulted in a disproportionate accumulation of proteins in the cell wall (CW) fraction, this likely reflects a rapid, destructive reaction at the cell surface that leads to gross structural collapse and the entrapment of intracellular components within insoluble debris. This “surface-level quenching” of NaClO explains why it requires significantly higher concentrations to achieve complete inactivation of NTM. In contrast, CAW maintained a normal distribution of protein mass across all fractions while inducing extensive high-molecular-weight smearing in the cytosolic and membrane (M) fractions (Figure 9B). This indicates that CAW effectively penetrates the robust mycobacterial cell wall to reach internal targets. The resulting massive aggregation of functional proteins—including those involved in the respiratory chain—is the direct cause of the irreversible collapse of membrane potential and the cessation of ATP production observed in this study.

NADH dehydrogenase, which is a large, multi-subunit enzyme forming part of the respiratory chain, is a major contributor to intracellular ROS [27]. We confirmed the reduction in ROS generation after treatment with CAW or PB-CAW, correlating with the reduction in membrane potential after treatment with these sanitizers (Figure 5). This irreversible loss of membrane potential and subsequent cessation of ATP production align with recent proteomic and biochemical findings in *Mycobacterium* species. Specifically, intense oxidative stress has been shown to directly target iron-sulfur clusters within internal respiratory enzymes, leading to a complete disruption of oxidative phosphorylation and metabolic collapse [28,29]. As mentioned above, CAW has been reported to preferentially react with cysteine and histidine. These amino acid residues are essential for the structures of protein complexes responsible for electron transfer in the respiratory chain, which contain cofactors such as heme and iron-sulfur clusters [30,31,32], supporting our conclusion that the bactericidal mechanism of CAW and PB-CAW on NTM involves membrane protein degeneration, especially the components of the respiratory chain.

While a systematic pH-dependency study was not the primary focus of this research, the consistent bactericidal efficacy observed between CAW (pH 5.0–6.0) and PB-CAW (pH 7.0–8.0) is noteworthy. In conventional NaClO solutions, the antimicrobial activity is known to be highly pH-dependent; as the pH increases toward neutral or alkaline conditions, the potent hypochlorous acid (HOCl) dissociates into the hypochlorite ion (OCl^−^), which possesses significantly lower membrane permeability. In contrast, our results showed that CAW and PB-CAW maintained high efficacy regardless of the pH difference between them. This may be attributed to the continuous generation of chloroperoxyl radicals (ClOO˙) within the solution. Unlike OCl^−^, these radical species are thought to maintain high permeability across the mycobacterial cell membrane independently of the environmental pH. The sustained presence of such reactive intermediates ensures that CAW can bypass the pH-dependent limitations of traditional chlorine-based disinfectants, allowing it to target internal functional proteins effectively even under neutralized clinical conditions.

Furthermore, the practical advantage of CAW is underscored by its superior safety profile. Although CAW induces potent protein aggregation in NTM, it exhibited significantly lower cytotoxicity toward human lung-derived A549 cells compared to the high-concentration NaClO typically required for mycobacterial disinfection (Figure 10). At 100 mg/L—the concentration sufficient for NTM eradication—CAW maintained over 50% cell viability, whereas 1000 mg/L NaClO caused severe cellular damage. This balance between high microbicidal efficacy through internal penetration and low overall toxicity makes CAW a promising candidate for environmental sanitation in clinical and domestic settings where NTM exposure is a concern.

A limitation of this study is that the results are obtained from in vitro analysis. While this study focused on planktonic cells, it is important to consider that NTM are notorious for forming robust biofilms in clinical and domestic plumbing systems, which significantly enhances their tolerance to chemical disinfectants. Given that historical reports have documented the emergence of glutaraldehyde-resistant NTM strains [33,34], the potential for resistance to any novel disinfectant must be carefully managed. Our findings demonstrate that CAW maintains its potent microbicidal efficacy even in the presence of high organic loads (Figure 1), suggesting its ability to bypass physical barriers. Furthermore, while our findings strongly suggest that CAW targets critical components of the respiratory chain, future proteomic studies—including mass spectrometry-based redox proteomics—are planned to identify the specific cysteine or histidine residues targeted by CAW-induced oxidation. Such analyses will provide a high-resolution understanding of how this protein aggregation triggers irreversible inactivation.

To mitigate the risk of resistance and ensure the eradication of biofilm-embedded NTM, it is recommended that CAW be used at sufficiently high concentrations (e.g., 100 mg/L) in practical settings. Future studies focusing on established NTM biofilms and the long-term impact of CAW exposure on microbial resistance will be essential to further validate its clinical utility.

Therefore, the unique oxidative mechanism of CAW, characterized by its high cellular penetration and subsequent induction of global protein aggregation, provides a promising strategic advantage for overcoming the inherent resistance and environmental persistence of NTM.

## 4. Materials and Methods

### 4.1. Bacterial Strains and Culture Conditions

The NTM strains used in this study are listed in Table S1. For all the strains, glycerol stocks stored at −80 °C were streaked onto Middlebrook 7H10 agar (Becton, Dickinson and Company, BD, Franklin Lakes, NJ, USA) and incubated at 37 °C for one week. The resulting colonies were picked and suspended in Middlebrook 7H9 liquid medium (Sigma-Aldrich), then cultured at 37 °C with shaking (130 rpm) for two weeks until sufficient growth was obtained.

### 4.2. Disinfectants

Chlorous acid water (CAW) and PB-CAW were obtained from Sankei Co., Ltd. (Osaka, Japan). Sodium hypochlorite (NaClO) was purchased from Oyalox Co. Ltd. (Tokyo, Japan). Free available chlorine (FAC) levels were measured by the method employing *N*,*N*-diethyl-*p*-phenylenediamine (DPD) according to the standard protocols. FAC levels are expressed in mg/L in this study. The FAC levels of the CAW stock solution and the prepared CAW were 2302 mg/L and 228 mg/L, with a pH of 4.22 and 6.18, respectively. We strictly controlled the experimental concentrations by diluting this stock solution with sterile distilled water. DPD was obtained from FUJIFILM Wako Pure Chemical Co. (Osaka, Japan).

### 4.3. Bactericidal Assays

Mycobacterial cultures (5.0 mL) were centrifuged (14,000× *g*, 4 °C, 5 min) and resuspended in 1.0 mL of saline solution with 0.05% Tween 20. Optical densities at 600 nm (OD_600_) of the individual cell suspensions were adjusted to 1.0 with saline/0.05% Tween 20. For the assays with organic-matter-free conditions, 0.9 mL of each of the disinfectants was mixed with 0.1 mL of bacterial suspension. In the case of organic-matter-load conditions, 0.1 mL of the bacterial inoculum, which was prepared by equally mixing 7.5% bovine serum albumin (BSA, Sigma-Aldrich, St. Louis, MO, USA) and the bacterial suspension (OD_600_ = 1.0), was added to 0.9 mL of each of the disinfectants. The treatment time at 25 °C was 1 min for both conditions. After treatment, 0.2 mL of 1 M sodium thiosulfate solution (FUJIFILM Wako Pure Chemical Co.) was immediately added to neutralize residual chlorine. After neutralization, 10-fold serial dilutions were prepared with saline/0.05% Tween 20. Appropriate dilutions (0.1 mL) were spread on Middlebrook 7H10 agar medium, and the plates were incubated at 37 °C for 4 weeks for *M. intracellulare* and 1 week for other mycobacteria tested. The number of surviving bacteria was measured by counting the colonies formed on the plates. As a control, the viability of the bacteria in pre-neutralized disinfectants was monitored using standard plating methods.

### 4.4. Scanning Electron Microscopy

*M. intracellulare* NBRC 112750 cells, which were treated with distilled water and 200 mg/L each of CAW or NaClO, were fixed overnight with 2% glutaraldehyde in cacodylate buffer (pH 7.4) at 4 °C. For scanning electron microscopy (SEM), each cell sample was dehydrated with a series of acetone solutions ranging in 10% increments from 50% (*v*/*v*) ethanol in distilled water to absolute acetone. All samples were dried to the critical point using a critical point dryer HCP-2 (Hitachi High-Technologies Corporation, Tokyo, Japan), coated with gold, and examined by SEM (Hitachi S-800; Hitachi High-Technologies Corporation).

### 4.5. Pulsed-Field Gel Electrophoresis (PFGE)

PFGE of *M. intracellulare* NBRC 112750 was conducted to assess the chromosomal degradation after treatment with CAW or NaClO according to the method described by Samir et al. [35]. In brief, *M. intracellulare* NBRC 112750 cells were collected by centrifugation (14,000× *g*, 5 min, 4 °C) from the reaction mixture after 30 min of treatment with saline, CAW or NaClO following neutralization. The pellet was washed with 500 μL of TE buffer (10 mM Tris-HCl, 1 mM EDTA, pH 8.0) and then resuspended in 200 μL of TE buffer. The sample plug molds, which were prepared with 200 μL of the cell suspension, were immersed in 4 mg/L lysozyme solution (Sigma Aldrich) and treated at 37 °C for 48 h (lysozyme solution was exchanged every 24 h). Subsequently, they were immersed in 0.5 M EDTA (Dojindo, Kumamoto, Japan) solution containing 2 mg/L proteinase K (FUJIFILM Wako Pure Chemical Co.) and 1% lauroyl sarcosine sodium (Nacalai Tesque, Kyoto, Japan), and the samples were statically incubated at 55 °C for 7 days (the solution was exchanged on the 5th day). The plugs were washed four times with TE buffer (each wash lasting 1 h). The obtained sample plugs containing DNA fragments were analyzed by pulsed-field gel electrophoresis (PFGE) using a CHEF-DR2 (Bio-Rad Laboratories, Hercules, CA, USA) under the following conditions: running buffer, 1× Tris-borate EDTA; running temperature, 14 °C; running time, 48 h; voltage gradient, 6 V/cm; switching time: 5 s to 35 s for 48 h.

### 4.6. DNA Extraction

After *M. intracellulare* NBRC 112750 cells were collected from the cultures by centrifugation (14,000× *g*, 5 min, 4 °C), pellets were suspended in 0.9 mL of TE buffer (pH 8.0) containing 4 μg/mL lysozyme and incubated at 37 °C for 1 h. Subsequently, 50 μL of 5.8 mg/mL achromopeptidase (FUJIFILM Wako Pure Chemical Co.) was added, and the reaction mixture was incubated at 37 °C for 1 h. Then, 110 μL of 20 μg/mL proteinase K was added, and the mixture was further incubated at 55 °C for 1 to 1.5 h. Next, 120 μL of 10% sodium dodecyl sulfate (FUJIFILM Wako Pure Chemical Co.) was added and incubated at 55 °C for 1 h. The DNA was extracted using phenol/chloroform/isoamyl alcohol (25:24:1, Nippon Gene, Toyama, Japan) followed by ethanol precipitation at −80 °C overnight. The extracted DNA was dissolved in TE buffer (pH 8.0) and stored at −20 °C until use.

### 4.7. Digestion of Single-Stranded DNA

To evaluate the amount of single-stranded DNA produced by treatment with CAW or NaClO, extracted bacterial DNAs were digested with S1 nuclease (Takara Bio, Kusatsu, Japan). The digested DNAs were electrophoresed in 0.8% Agarose S (Nippon Gene), and DNA fragmentation was evaluated using ethidium bromide (Thermo Fisher Scientifi Inc., Waltham, MA, USA) staining of the gels.

### 4.8. Measurement of Membrane Potential

After bactericidal assays were performed (treatment time 1 min) as described above, *M. intracellulare* NBRC 112750 cells were collected by centrifugation (14,000× *g*, 4 °C, 5 min), suspended in 1 mL of Middlebrook 7H9 medium supplemented with ADC (0.2% (*w*/*v*) dextrose, 0.2% (*v*/*v*) glycerol and 0.5% bovine serum albumin) and incubated at 37 °C for 30 min. As a control, we heated an *M. intracellulare* NBRC 112750 cell suspension at 100 °C for 10 min and filtered the suspension through a 40 μm cell strainer (Corning Inc., Corning, NY, USA). We also prepared carbonyl cyanide *m*-chlorophenyl hydrazone (CCCP)-treated *M. intracellulare* NBRC 112750 cells as a control. Membrane potentials of the *M. intracellulare* NBRC 112750 cells after each treatment were measured using BacLight™ Bacterial Membrane Potential Kit (Fisher Thermo Scientific, Inc.) according to a previous report [36]. Green and red fluorescence were measured using a flow cytometer (Merck Millipore, Burlington, MA, USA), and the changes in membrane potential were evaluated based on the ratio of red fluorescence to green fluorescence of the stained cells.

### 4.9. Measurement of ATP

ATP levels in *M. intracellulare* NBRC 112750 cultures were measured according to the method reported by Yuroff et al. [37]. Cell suspensions of *M. intracellulare* NBRC 112750 were adjusted to an OD_600_ of 1.0 with saline/0.5% Tween 20. Next, 0.1 mL of the cell suspension was mixed with 0.9 mL of the test reagent (H_2_O, CAW, PB-CAW or NaClO) and incubated at room temperature for 1 min. Subsequently, residual chlorine was neutralized with 0.2 mL of 1 M sodium thiosulfate. The treated cells were collected by centrifugation (14,000× *g*, 4 °C, 5 min) and resuspended in 1 mL of Middlebrook 7H9 medium supplemented with ADC. The cell suspension was diluted 10-fold with the same medium and cultured at 37 °C for pre-scheduled intervals. The culture (0.5 mL) was periodically sampled and frozen at −80 °C for 30 min. The samples were then thawed at room temperature to lyse the cells, and 50 μL of each cell lysate was dispensed into a 96-well plate. Sterilized distilled water (dH_2_O) and 50 μL of BacTiter-Glo™ reagent (Promega, Madison, WI, USA) were added and statically reacted at room temperature for 5 min. The luminescence intensity was measured using a GloMax^®^ Navigator System (Promega). The obtained luminescence values were evaluated as an indicator of surviving bacterial cells based on the amount of ATP.

### 4.10. ROS Measurement

The reactive oxygen species (ROS) that were generated in *M. intracellulare* NBRC 112750 after treatment with CAW or NaClO were measured using the OxiSelect™ Intracellular ROS Assay Kit (Cell Biolabs Inc., San Diego, CA, USA) according to the manufacturer’s instructions. In brief, *M. intracellulare* NBRC 112750 cell suspensions were adjusted to an OD_600_ of 2.0 with Middlebrook 7H9 medium supplemented with ADC, then centrifuged (14,000× *g* at 4 °C, 5 min) and resuspended in 200 μL of a 1× solution of dichlorofluorescein-diacetate (DCFH-DA) (Thermo Fisher Scientific, Inc.). After the reaction mixture was incubated at 37 °C for 1 h in the dark, the cells were collected by centrifugation (14,000× *g*, 4 °C, 5 min) and resuspended in 2 mL of 1× PBS (pH 7.4). Then, 100 μL of each test reagent (CAW or NaClO) was dispensed into a 96-well plate and mixed with the same volume of the bacterial suspension. Hydrogen peroxide (1 mM and 10 mM) was used as a positive control. Immediately after mixing, changes in fluorescence intensity due to oxidative stress were recorded at 1 min intervals for 1 h. Fluorescence was monitored at an excitation wavelength of 480 nm and an emission wavelength of 530 nm using a fluorometer (Corona Electric Co., Ltd., Hitachinaka, Japan).

### 4.11. Subcellular Fractionation and Protein Extraction for Target Identification

To investigate the impact of disinfectants on bacterial constituent proteins and to identify the specific targets of CAW, *M. intracellulare* cells were subjected to subcellular fractionation. The bacterial suspension (OD_600_ = 8.0) was treated with CAW, NaClO, or sterile water for 1 min, followed by immediate neutralization with 1 M sodium thiosulfate. The harvested cells were washed with PBS and resuspended in a protease inhibitor cocktail to prevent proteolytic degradation during processing. The suspension was then subjected to mechanical disruption via sonication (30 min, 30 s ON/OFF cycles). To isolate and define the target compartments, the lysate was centrifuged at 20,000× *g* for 20 min at 4 °C. The resulting pellet, containing insoluble cell wall components and associated proteins, was collected as the cell wall (CW) fraction. To evaluate morphological changes, the isolated CW fraction was Gram-stained using the Favor G kit (Nissui Pharmaceutical Co., Ltd., Tokyo, Japan) and observed under a light microscope. The supernatant was further processed by ultracentrifugation at 150,000× *g* for 90 min at 4 °C. The final supernatant, containing soluble intracellular proteins, was collected as the cytosol (CS) fraction, while the resulting pellet, representing the cytoplasmic membrane components, was designated as the M fraction.

### 4.12. SDS-PAGE and Silver Staining for Protein Profile Analysis

To visually assess the effects of CAW on bacterial proteins and identify potential targets, SDS-PAGE was performed on the isolated fractions. First, the protein concentration of each subcellular fraction (CW, CS, and M) was quantified using the Pierce™ BCA Protein Assay Kit (Thermo Fisher Scientific, Inc.). Each fraction was then mixed at a 1:1 (*v*/*v*) ratio with a sample buffer consisting of 95 μL of 2× SDS loading buffer and 5 μL of 2-mercaptoethanol (Sigma-Aldrich). To ensure complete protein denaturation, the mixtures were heated at 100 °C for 5 min, followed by cooling to room temperature and brief centrifugation. A 5 μL aliquot of each denatured sample was loaded onto a Mini-PROTEAN TGX Gel (Bio-Rad Laboratories), and electrophoresis was conducted at a constant voltage of 200 V for 30 min using a Mini-PROTEAN Tetra Vertical Electrophoresis Cell (Bio-Rad Laboratories) powered by a PowerPac Universal Power Supply (Bio-Rad Laboratories). Following electrophoresis, the gels were silver stained using the SilverQuest Silver Staining Kit (Invitrogen, Thermo Fisher Scientific, Inc.) according to the manufacturer’s Fast protocol for visual observation of protein bands and potential smearing patterns, which served as indicators of protein aggregation.

### 4.13. Cytotoxicity Assay Using Human Lung-Derived A549 Cells

To evaluate the safety of CAW for environmental applications, cytotoxicity assays were performed using the A549 human lung carcinoma cell line (a model for Type II alveolar epithelial cells). A549 cells were cultured in Dulbecco’s Modified Eagle Medium (DMEM) supplemented with 10% fetal bovine serum (FBS) and 1% penicillin-streptomycin at 37 °C in a humidified atmosphere containing 5% CO_2_. For the cytotoxicity test, the cells were seeded into 24-well plates and grown to subconfluence. The culture medium was removed, and the cells were washed once with phosphate-buffered saline (PBS). The cells were then treated with 500 μL of CAW or PB-CAW at varying FAC concentrations (5, 10, 25, 50, and 100 mg/L) for 1 min at room temperature. Sterile water and 1000 mg/L NaClO were used as negative and positive cytotoxic controls, respectively. Immediately following the 1 min treatment, the solutions were removed, and the cells were washed with PBS and then detached using a trypsin-EDTA solution. Cell viability and total cell counts were determined using the trypan blue exclusion method. Briefly, the cell suspension was mixed with an equal volume of 0.4% trypan blue solution, and the number of viable (unstained) and dead (blue-stained) cells was counted using a hemocytometer (C-Chip; NanoEnTek Inc., Seoul, Republic of Korea). The survival rate (%) was calculated as the percentage of viable cells relative to the total cell count, and all experiments were performed in triplicate to calculate the mean ± standard error.

### 4.14. Statistical Analysis

All statistical analyses were performed using R software (version 4.4.2). A *p*-value of <0.05 was considered to be statistically significant unless otherwise noted. Primary statistical analyses were conducted using a one-way analysis of variance (ANOVA) followed by Tukey’s test or Dunnett’s test. For the membrane potential assays, comparisons of the fluorescence ratios were performed using two-sample *t*-tests.

## 5. Conclusions

CAW was effective in killing NTM even under conditions that are rich in organic matter. This superior efficacy is driven by a unique mechanism: CAW penetrates the mycobacterial cell wall to induce extensive aggregation of internal functional proteins, leading to the rapid collapse of membrane potential and ATP depletion. Furthermore, CAW exhibits lower genotoxicity and significantly lower cytotoxicity toward human lung cells compared to NaClO. Consequently, CAW and PB-CAW are highly effective and safer sanitizers that can decrease the NTM load in wet environments, thereby reducing the risk of NTM exposure and lung disease in susceptible individuals.

## Data Availability

The original contributions presented in this study are included in the article/Supplementary Materials. Further inquiries can be directed to the corresponding author.

## Supplementary Materials

The following supporting information can be downloaded at: https://www.mdpi.com/article/10.3390/ijms27104570/s1.

## Abbreviations

The following abbreviations are used in this manuscript:

ATP	Adenosine triphosphate
BCA	Bicinchoninic acid
BSA	Bovine serum albumin
CAW	Chlorous acid water
CCCP	Carbonyl cyanide *m*-chlorophenyl hydrazone
CS	Cytosolic fraction
CW	Cell wall fraction
FAC	Free available chlorine
M	Membrane fraction
MAC	*Mycobacterium avium* complex
NaClO	Sodium hypochlorite
NTM	Non-tuberculous mycobacteria
PB-CAW	Phosphate-buffered chlorous acid water around neutral pH
PFGE	Pulsed-field gel electrophoresis
ROS	Reactive oxygen species
SDS-PAGE	Sodium dodecyl sulfate–polyacrylamide gel electrophoresis

## References

[1] Ministry of Health, Labour and Welfare (2024). Annual Report on Tuberculosis Registrant Information Survey 2023.

[2] Namkoong H., Kurashima A., Morimoto K., Hoshino Y., Hasegawa N., Ato M., Mitarai S. (2016). Epidemiology of Pulmonary Nontuberculous Mycobacterial Disease, Japan. Emerg. Infect. Dis..

[3] Winthrop K.L., McNelley E., Kendall B., Marshall-Olson A., Morris C., Cassidy M., Saulson A., Hedberg K. (2010). Pulmonary Nontuberculous Mycobacterial Disease Prevalence and Clinical Features: An Emerging Public Health Disease. Am. J. Respir. Crit. Care Med..

[4] Matsuyama M., Matsumura S., Nonaka M., Nakajima M., Sakai C., Arai N., Ueda K., Hizawa N. (2023). Pathophysiology of Pulmonary Nontuberculous Mycobacterial (NTM) Disease. Respir. Investig..

[5] Shah N.M., Davidson J.A., Anderson L.F., Lalor M.K., Kim J., Thomas H.L., Lipman M., Abubakar I. (2016). Pulmonary *Mycobacterium avium-intracellulare* Is the Main Driver of the Rise in Non-Tuberculous Mycobacteria Incidence in England, Wales and Northern Ireland, 2007–2012. BMC Infect. Dis..

[6] Daley C.L. (2017). *Mycobacterium avium* Complex Disease. Microbiol. Spectr..

[7] Morimoto K., Hasegawa N., Izumi K., Namkoong H., Uchimura K., Yoshiyama T., Hoshino Y., Kurashima A., Sokunaga J., Shibuya S. (2017). A Laboratory-Based Analysis of Nontuberculous Mycobacterial Lung Disease in Japan from 2012 to 2013. Ann. Am. Thorac. Soc..

[8] Morimoto K., Ato M., Hasegawa N., Mitarai S. (2021). Population-Based Distribution of *Mycobacterium avium* and *Mycobacterium intracellulare* in Japan. Microbiol. Res..

[9] van Ingen J., Obradovic M., Hassan M., Lesher B., Hart E., Chatterjee A., Daley C.L. (2021). Nontuberculous Mycobacterial Lung Disease Caused by *Mycobacterium avium* Complex—Disease Burden, Unmet Needs, and Advances in Treatment Developments. Expert Rev. Respir. Med..

[10] Koh W.-J., Jeong B.-H., Jeon K., Lee N.Y., Lee K.S., Woo S.Y., Shin S.J., Kwon O.J. (2012). Clinical Significance of the Differentiation between *Mycobacterium avium* and *Mycobacterium intracellulare* in *M. avium* Complex Lung Disease. Chest.

[11] Griffith D.E., Aksamit T., Brown-Elliott B.A., Catanzaro A., Daley C., Gordin F., Holland S.M., Horsburgh R., Huitt G., Iademarco M.F. (2007). An official ATS/IDSA statement: Diagnosis, treatment, and prevention of nontuberculous mycobacterial diseases. Am. J. Respir. Crit. Care Med..

[12] Wallace R.J., Brown-Elliott B.A., McNulty S., Philley J.V., Killingley J., Wilson R.W., York D.S., Shepherd S., Griffith D.E. (2014). Macrolide/Azalide therapy for nodular/bronchiectatic *Mycobacterium avium* complex lung disease. Chest.

[13] Lee B.Y., Kim S., Hong Y., Lee S.-D., Kim W.S., Kim D.S., Shim T.S., Jo K.-W. (2015). Risk factors for recurrence after successful treatment of *Mycobacterium avium* complex lung disease. Antimicrob. Agents Chemother..

[14] Nishiuchi Y., Iwamoto T., Maruyama F. (2017). Infection Sources of a Common Non-Tuberculous Mycobacterial Pathogen, *Mycobacterium avium* Complex. Front. Med..

[15] Whiley H., Keegan A., Giglio S., Bentham R. (2012). *Mycobacterium avium* Complex—The Role of Potable Water in Disease Transmission. J. Appl. Microbiol..

[16] Nishiuchi Y., Maekura R., Kitada S., Tamaru K., Taguri T., Kira Y., Hiraga T., Hirotani A., Yoshimura K., Miki M. (2007). The recovery of *Mycobacterium avium-intracellulare* complex (MAC) from the residential bathrooms of patients with pulmonary MAC. Clin. Infect. Dis..

[17] Dantec C.L., Duguet J.-P., Montiel A., Dumoutier N., Dubrou S., Vincent V. (2002). Chlorine disinfection of atypical mycobacteria isolated from a water distribution system. Appl. Environ. Microbiol..

[18] Taylor R.H., Falkinham J.O., Norton C.D., LeChevallier M.W. (2000). Chlorine, chloramine, chlorine dioxide, and ozone susceptibility of *Mycobacterium avium*. Appl. Environ. Microbiol..

[19] Goda H., Nakayama-Imaohji H., Yamaoka H., Tada A., Nagao T., Fujisawa T., Koyama A.H., Kuwahara T. (2022). Inactivation of Human Norovirus by Chlorous Acid Water, a Novel Chlorine-Based Disinfectant. J. Infect. Chemother..

[20] Ministry of Health, Labour and Welfare (2018). The 9th Edition of the Japanese Standards for Food Additives.

[21] eCFR 21 CFR 173.325—Acidified Sodium Chlorite Solutions. https://www.ecfr.gov/current/title-21/chapter-I/subchapter-B/part-173/subpart-D/section-173.325.

[22] Hatanaka N., Awasthi S.P., Goda H., Kawata H., Hinenoya A., Yamasaki S. (2025). Chlorous Acid Inactivates *Mycobacterium tuberculosis* with Much Lower Available Chlorine Concentration than Sodium Hypochlorite. Jpn. J. Infect. Dis..

[23] Ichii O., Nakamura T., Hiraishi M., Namba T., Rubel M.Z.U., Umeyama T., Asai M. (2025). Application of chlorous acid water for disinfection of surgical site in dairy cows. Front. Vet. Sci..

[24] Kawata H., Kohno M., Nukina K., Horiuchi I., Goda H., Kuwahara T., Yoshimori K., Miyaji A., Kamachi T., Yoshikawa T. (2021). Identifying the Chloroperoxyl Radical in Acidified Sodium Chlorite Solution. PLoS ONE.

[25] Gupta A., Imlay J.A. (2022). *Escherichia coli* Induces DNA Repair Enzymes to Protect Itself from Low-Grade Hydrogen Peroxide Stress. Mol. Microbiol..

[26] Hatanaka N., Awasthi S.P., Goda H., Kawata H., Uchino Y., Kubo T., Aoki S., Hinenoya A., Yamasaki S. (2020). Chlorous Acid Is a More Potent Antibacterial Agent than Sodium Hypochlorite against *Campylobacter*. Food Control.

[27] Zhao R.-Z., Jiang S., Zhang L., Yu Z.-B. (2019). Mitochondrial Electron Transport Chain, ROS Generation and Uncoupling (Review). Int. J. Mol. Med..

[28] Tripathi A., Anand K., Das M., O’Niel R.A., Sabarinath P.S., Thakur C., Reddy R.R.L., Rajmani R., Chandra N., Laxman S. (2022). *Mycobacterium tuberculosis* requires SufT for Fe-S cluster maturation, metabolism, and survival in vivo. PLoS Pathog..

[29] Elchennawi I., Ollagnier de Choudens S. (2022). Iron–sulfur clusters toward stresses: Implication for understanding and fighting tuberculosis. Inorganics.

[30] Xia D., Yu C.A., Kim H., Xia J.Z., Kachurin A.M., Zhang L., Yu L., Deisenhofer J. (1997). Crystal Structure of the Cytochrome *bc1* Complex from Bovine Heart Mitochondria. Science.

[31] Devlin T., Hofman C.R., Acevedo Z.P.V., Kohler K.R., Tao L., Britt R.D., Hoke K.R., Hunsicker-Wang L.M. (2019). DEPC Modification of the CuA Protein from *Thermus thermophilus*. J. Biol. Inorg. Chem..

[32] Snyder C.H., Merbitz-Zahradnik T., Link T.A., Trumpower B.L. (1999). Role of the Rieske Iron-Sulfur Protein Midpoint Potential in the Protonmotive Q-Cycle Mechanism of the Cytochrome *bc1* Complex. J. Bioenerg. Biomembr..

[33] Griffiths P.A., Babb J.R., Fraise A.P. (1997). Glutaraldehyde-resistant *Mycobacterium chelonae* from endoscope washer disinfectors. J. Appl. Microbiol..

[34] Walsh S.E., Maillard J.-Y., Russell A.D., Hann A.C. (2001). Possible mechanisms for the relative efficacies of *ortho*-phthalaldehyde and glutaraldehyde against glutaraldehyde-resistant *Mycobacterium chelonae*. J. Appl. Microbiol..

[35] Singh S.P., Salamon H., Lahti C.J., Farid-Moyer M., Small P.M. (1999). Use of Pulsed-Field Gel Electrophoresis for Molecular Epidemiologic and Population Genetic Studies of *Mycobacterium tuberculosis*. J. Clin. Microbiol..

[36] Chawla M., Singh A. (2013). Detection of Membrane Potential in *Mycobacterium tuberculosis*. Bio-protocol.

[37] Yuroff A., Fan F., Butler B., Collins M. (2008). Application of the BacTiter-Glo™ Assay for Rapid Enumeration and Screening of Antimicrobial Compounds for *Mycobacterium avium* Complex Bacteria. Promega Notes.

