# Supplementary material for: Bactericidal Mechanism of Chlorous Acid Water in the Inactivation of Non-Tuberculous Mycobacteria
Source: Int J Mol Sci. 2026 May 19;27(10):4570. doi: 10.3390/ijms27104570 (PMC13207738; doi:10.3390/ijms27104570)
Supplement: Supplementary file 1 [file ijms-27-04570-s001.zip › ijms-4154774-supplementary.pdf]

Table S1. NTM strains used in this study.

Strain	Species	Source
NBRC 112750	*Mycobacterium intracellulare*	Human tracheal lavage fluid
JCM 6379	*Mycobacterium kansasii*	Human inguinal sinus, Kansas City, MO, USA.
JCM 6387	*Mycobacterium fortuitum*	Cold abscess of human
JCM 6390	*Mycobacterium chelonae*	Sputum of a patient with pulmonary lesion
JCM 13569	*Mycobacterium abscessus*	Human knee abscess
JCM 15429	*Mycobacterium avium*	Bathroom of a patient with pulmonary *Mycobacterium avium* complex disease
JCM 15430	*Mycobacterium avium*	Sputum of a patient with pulmonary *Mycobacterium avium* complex disease
JCM 15431	*Mycobacterium avium*	Bathroom of a patient with pulmonary *Mycobacterium avium* complex disease
JCM 15432	*Mycobacterium avium*	Sputum of a patient with pulmonary *Mycobacterium avium* complex disease

NTM, none-tuberculous mycobacteria

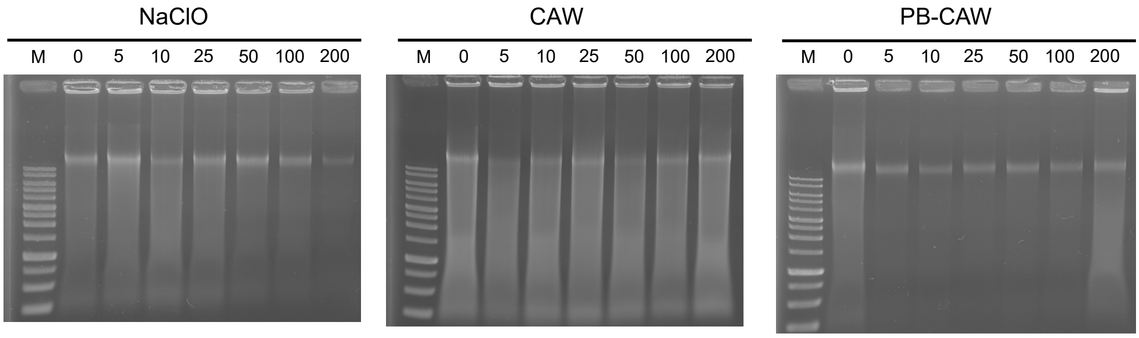

Figure S1. Agarose gel electrophoresis of the DNA extracted from *M. intracellulare* after 1-min treatment with indicated reagents. The numbers above the images indicate the free available chlorine concentration in the reaction mixture. M, 1-kb DNA ladder marker.

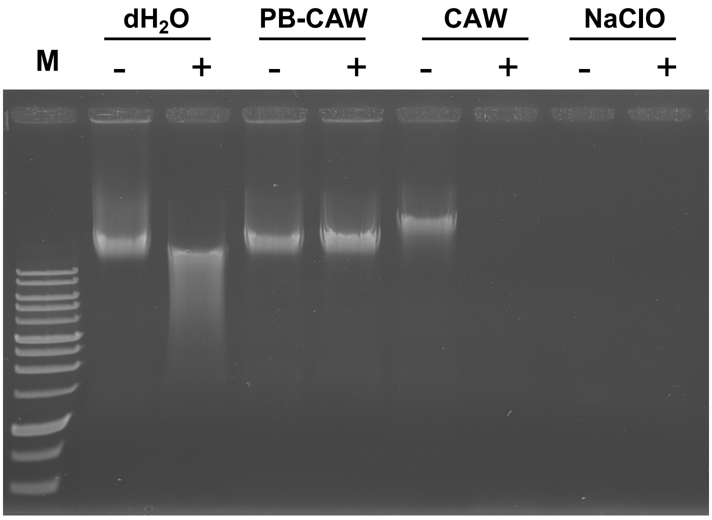

Figure S2. Agarose gel electrophoresis of *M. intracellulare* DNA after treatment with/without S1 nuclease. *M. intracellulare* NBRC112750 cell was treated with 200 mg/L each of the reagent for 30 min. After neutralization of FAC, the DNA was extracted and treated with S1 nuclease at 37°C for 1 h. -, without S1 nuclease; +, with S1 nuclease; M, 1-kb DNA ladder marker.
